# A novel technique to develop thoracic spinal laminectomy and a methodology to assess the functionality and welfare of the contusion spinal cord injury (SCI) rat model

**DOI:** 10.1371/journal.pone.0219001

**Published:** 2019-07-02

**Authors:** Harikrishnan V. S., Lissy K. Krishnan, Klas S. P. Abelson

**Affiliations:** 1 Division of Laboratory Animal Science, Department of Applied Biology, Biomedical Technology Wing, Sree Chitra Tirunal Institute for Medical Sciences and Technology, Thiruvananthapuram, Kerala, India; 2 Department of Experimental Medicine, Faculty of Health and Medical Sciences, University of Copenhagen, Copenhagen, Denmark; 3 Division of Thrombosis Research, Department of Applied Biology, Bio Medical Technology Wing, Sree Chitra Tirunal Institute for Medical Sciences and Technology, Thiruvananthapuram, Kerala, India; Uniformed Services University, UNITED STATES

## Abstract

This study reports the advantage of a novel technique employing a motorised dental burr to assist laminectomy over the conventional manual technique at T10-T11 vertebra level in a rat model of spinal cord injury. Twenty-four female rats were randomly assigned to four groups: (1) conventionally laminectomised, (2) dental burr assisted laminectomised, (3) conventionally laminectomised with spinal cord contusion and (4) dental burr assisted laminectomised with spinal cord contusion. Basso Beattie Bresnahan (BBB) score, postoperative body weights, rat grimace scale (RGS), open cage activity and rearing was studied at 1, 7, 14, 21 and 28 days postoperatively, and area of spinal tissue affected was evaluated histologically. Laminectomised and spinal cord injured rats from dental burr groups showed significantly more weight gain and less weight loss respectively in comparison with respective conventionally laminectomised groups at various time points. Significantly higher RGS score was noticed in conventionally laminectomised animals on Day 1 in comparison to burr assisted laminectomy and presence of pain was evident until Day 7 in the conventionally spinal cord injured group. BBB score did not differ between techniques, whereas laminectomy groups showed more resting time than spinal injury groups. High rearing score was significantly higher in groups which underwent dental burr assisted technique at various time points with respect to their conventional counterparts. This study suggests that the use of dental burr assisted technique to perform laminectomy will bring refinement by producing less pain, aiding in better recovery, removing procedural artefacts without affecting the outcome of the model.

## Introduction

Spinal cord injury (SCI) research predominantly focuses on pathogenesis and development of treatment modalities and to hasten the recovery of patients. Rats are widely used in SCI research [1,2,3,4] and this species has aided in the development of a plethora of treatment methodologies which have undergone or are undergoing clinical trials [5]. The majority of injuries in humans occur at the cervical level [6]. However, owing to the severe complications associated with animals modelled with the cervical spinal damage [7], mid or low thoracic SCI remains the most commonly used site of injury in spinal research by most laboratories [7], with contusion injury being the most common type of injury [8].

Even though many devices and modified techniques have been introduced for spinal fixation and for inducing the SCI, such as NYU/MASCIS (New York University, Multicenter Animal Spinal Cord Injury Study) impactor and OSU (Ohio State University electromagnetic spinal cord injury device) impactor [9] there are no validated studies on alternative techniques that could be of potential advantage over the conventional technique to create the spinal laminectomy. Laminectomy in rodents could cause unintended trauma to the spinal cord, especially towards the anterior part of the axial skeleton where the vertebrae are in close proximity to the failed cord, which could be a cause for potential intervention to the study design. This could be a cause of concern where multiple surgeries are continuously carried out as part of study design where the surgeon solely depends upon manual skills and non-motorised equipment to perform laminectomy. Even though laminectomy is a widely performed technique, the surgical description, complications involved and possible alternatives to refine laminectomy in animal models are often overlooked and seldom reported. There are clear complications after conventional laminectomy and the subsequent bleedings in rat models. The complications are of such magnitude that they can actually be used modeling conditions like Failed Back Syndrome [10] and arachnoiditis [11].

The BBB (Basso Beattie and Bresnahan) test [12] is probably the most commonly used test of locomotor function in spinal cord injured rats worldwide [13]. A review of previous studies for a period of twelve years shows that BBB test serves as the first choice among behavioural testing methods for different types of thoracic spinal cord injuries including contusion [13]. BBB test can be considered as a gold-standard for spinal cord injury studies, as it is highly reliable and has been shown to provide reproducible results [14]. However, a very important drawback of the system is that the BBB score is ordinal, and not linear. The lower part of the scale concerns gross aspects of locomotion, while the upper part of the scale includes rather discrete movement aspects that do not represent major improvements in the animal’s motor ability [15]. Many modifications of the BBB scoring have been introduced thereafter, but most of them focused on functionality assessment based on motor tests [16].

The aim of the present study was to introduce and test a novel technique to create a thoracic (T10-T11) laminectomy assisted with a motorised dental-burr and compare it with the conventional technique. Welfare assessment is sparsely reported when modeling spinal cord injury studies in rats. Hence, it was decided to select and monitor a battery of welfare parameters while establishing an alternative surgical technique to develop SCI. The selected parameters were also used to compare the novel technique with the conventionally used procedure. Hence, the study focused to document a series of welfare parameters on laminectomised and SCI induced rats. The presence and duration of pain was attempted to be assessed which could be of value in determining the analgesic protocol for similar studies in the future. Therefore, in addition to the BBB scores, post-operative body weights, activity levels and rearing scores, Rat Grimace Scale (RGS) [17] and histopathology scores were also monitored to assess whether measures other than motor tests could be useful to validate the welfare and the outcome of research using rat SCI model.

## Materials and methods

### Ethical approval

The work with animals and their care was done in adherence with the “Breeding of and Experiments on Animals (Control and Supervision) Rules, 1998” under guidelines issued by CPCSEA, (Committee for the Purpose of Control and Supervision of Experiments on Animals), Government of India and the work was sanctioned by the Institutional Animal Ethics Committee.

### Animals and husbandry

The animals were supplied by Charles River (Hylasco Biotechnology, India). At the point of initiation of the study, animals were 9–12 weeks of age and weighed 240–280 grams. Health monitoring was done as per FELASA guidelines [18]. Animals were individually housed from one week prior to the day of surgery and were subjected and acclimatized to routine handling and gentling for 10 minutes, twice daily. Individually Ventilated Caging system (Citizen Industries, Ahmedabad, India) was used to house the animals in polysulfone cages of dimensions 800 cm^2^ and height of 18.5 cm with stainless steel 304 wire grill tops. Sterile corncob (Sparconn, Bangalore, India) was used as bedding material and the animals were fed *ad libitum* with pelleted feed (Safe rodent diet-D131, Augy, France) and filtered potable water was supplied in drinking bottles throughout the acclimatization and study periods. The environment in the animal facility as well as the surgical facility and observation areas were maintained at 22±2°C and the relative humidity between 30–70%. A 12–15 air changes/hour was maintained inside the animal room. The facility had an automated lighting which maintained a 12:12 light:dark photoperiodicity, and had a calibrated light intensity measurement record where the intensity of light was maintained below 325 Lux at 1 meter height from the floor level inside the animal room. All the procedures including the handling, gentling and acclimatization in RGS assessment box and open field maze, surgical procedures, body weight measurements, post-operative behavioural scores and functional assessments in animals were carried out between 9.00 am and 3.00 pm, by qualified and trained personnel throughout the entire duration of the study.

### Study design

A total of 24 Crl:WI female rats were used. Many of the previous publications indicated the use of female animals owing to the relative ease of manual bladder emptying after SCI induction resulting in less frequent urinary tract infections^3^ and so the present work was done with female rats. The animals for the study were randomised and grouped using the online research randomiser software (https://www.randomizer.org/). The animals were divided into four groups with six animals in each group. Group I consisted of animals which underwent laminectomy without SCI using the conventional surgical technique (CONV-LAM). Group II consisted of animals designated to undergo laminectomy without SCI using motorised dental burr assisted technique (DBA-LAM). In groups III and IV, animals underwent SCI performed by a conventional approach (CONV-SCI) and by a motorised dental burr assisted (DBA-SCI) approach respectively.

### Anaesthesia, surgical site preparation and radiographic confirmation of T10 vertebra

The rats were anaesthetised using 5mg/kg bodyweight xylazine (Xylaxin, Indian Immunologicals, Hyderabad, India) and 80mg/kg bodyweight ketamine (Aneket, Neon Laboratories limited, Thane, India) mixed in an insulin syringe and administered by intraperitoneal injection. Once the animals lost their righting and pedal pinch reflexes, the dorsum was clipped using a hair clipper (Oster, Golden A5, McMinnville, TN, USA) and the T10 vertebrae was identified by counting from the last rib level cranially. The exact position of T10 vertebra was confirmed using a metallic paper clip marked with identification ink at the level identified, where after a radiograph was obtained with reference to the mark previously made. The dorsum was prepared aseptically using povidone iodine solution and the animal was draped with sterile surgical drapes and positioned for surgery. 1.5–2% isofluorane (Forane, Abott India Limited, Mumbai, India) in oxygen (0.5–1 l/min) was administered through a precision vaporizer and face mask (E-Z system corporation, Palmer, PA) to maintain sufficient anesthesia and oxygenation during the surgery. Eye ointment (Neosporin, GlaxoSmithKline pharmaceuticals Ltd. Mumbai, India) was applied over the eyes of all animals immediately after the induction of anaesthesia.

### Conventional dorsal laminectomy

A skin incision of about 2.5 cm was made above the T10-T11 vertebrae as confirmed by radiography, and the paravertebral musculature was carefully incised to expose the vertebra. The vertebral spinous process was carefully removed using a micro rongeur, followed by exposure of the intact spinal cord by careful removal of the dorsal lamina using micro rongeurs and fine mosquito forceps. The total time of surgery from the start of skin incision until the completion of last skin closure was recorded and the length and width of laminectomy made was measured using a digital vernier caliper.

### Motorised dental burr assisted (DBA) dorsal laminectomy

**Fig 1:**
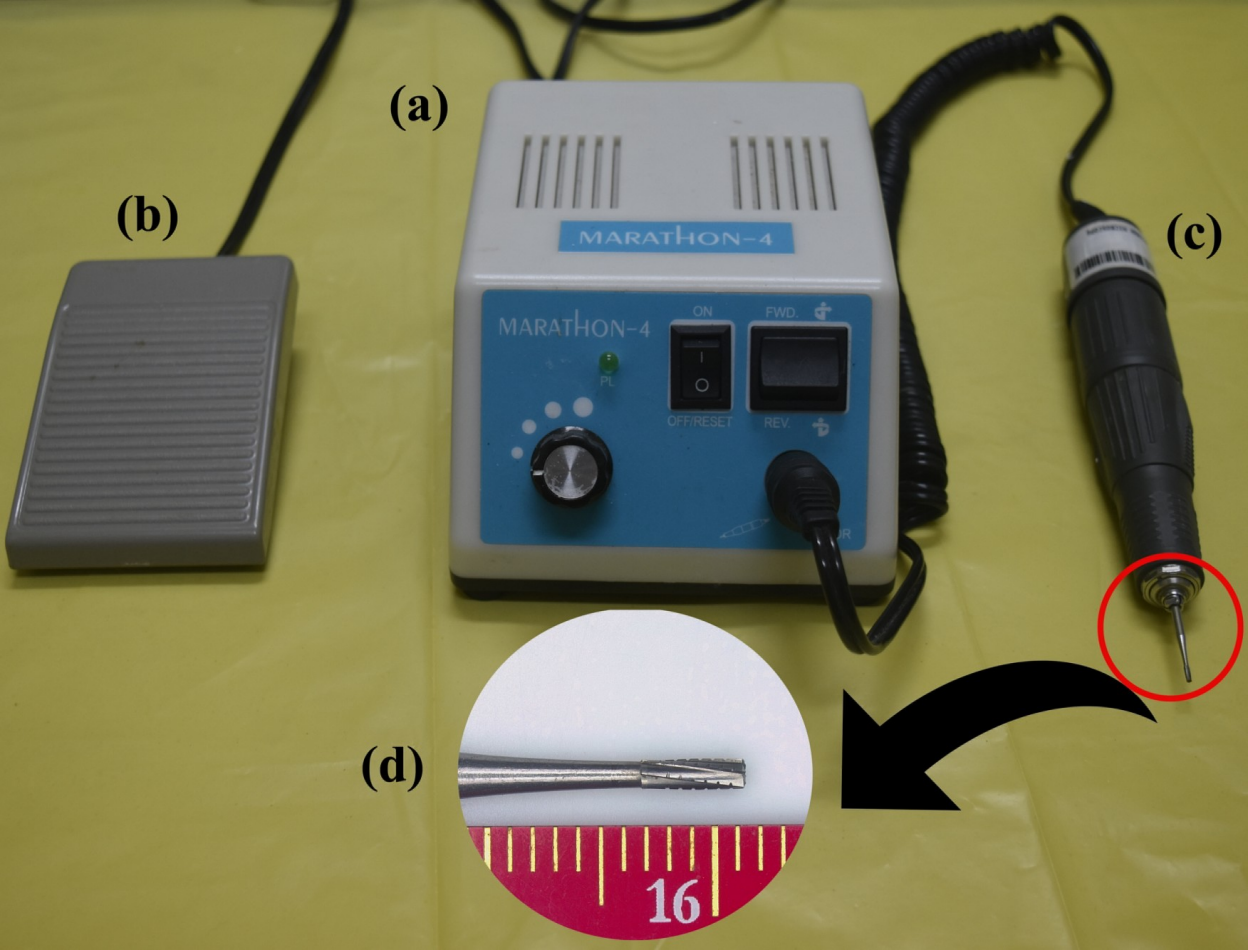
Motorised dental burr with its various components. Picture of the micromotor (Marathon-4, Maximum RPM-35000, SDE-H37LI, Saeyang Microtech, Korea) to power the dental burr assisted laminectomy (a) with pedal switch for operation (b). The sterile hand piece with attached replaceable dental burr (SS White Carbide burrs, SSWHP-559, NJ, USA) (c) and a magnified view of the dental burr with scale (d).

A skin incision of about 2.5 cm was made above the T10-T11 vertebrae as confirmed by radiography, and the paravertebral musculature was carefully incised to expose the vertebra. The vertebral spinous process was carefully removed using a micro rongeur. Using a dental burr (Carbide burrs, SSWHP-559, NJ, USA) equipped with a micromotor (Marathon-4, max RPM-35000, SDE-H37LI, Saeyang Microtech, Korea) operated using a pedal switch (Fig 1), the dorsal lamina was drilled on both sides. Care was taken not to cut through the entire depth of the dorsal lamina and thereby preventing injury to the spinal cord by the procedure. Sterile isotonic saline was dropped slowly while drilling to avoid thermal injury to the nearby tissues. The drilling was done into about one third of the entire thickness on the dorsal lamina. This became evident by feeling the free movement of the dorsal lamina on gentle touches with the tip of a fine jeweller’s forceps. At this stage, the dorsal lamina of the vertebra was totally removed using a fine jeweller’s forceps and then the intact spinal cord could be viewed. The total time of surgery from the start of skin incision until the completion of last skin closure was recorded and the length and width of laminectomy made was measured using a digital vernier caliper.

### Spinal cord injury (SCI)

Using a custom made 2.5 mm impounder tip, equipped with a spring-loaded force deliverer, a pre-calibrated force of 200 kilodynes (Kdyn) was delivered to the exposed spinal tissue. The injury of the spinal cord was confirmed by the observation of an immediate area of hematoma over the spinal cord tissue and a nervous twitch over the entire hind limb and tail region. The animals were further assessed on Day 1 postoperatively, to confirm an intended severe level of SCI at a BBB score of 0.

### Postoperative care

Animals with SCI invariably exhibited urine retention and urinary bladder distension. Manual evacuation of urine was therefore performed in the animals until the animals regained control to perform normal micturition. A few animals in SCI group could not defecate during the first post-operative day and manually assisted voiding of pellets was performed. Clinical signs of welfare in all the four groups of animals were assessed in terms of presence of porphyrin stained tears, grooming, activity levels, food intake and water consumption. The animals were provided with two wheat biscuits (Britannia Tiger Glucose Cookie, 5/1A Hungerford Street, Kolkata, India), and 5 g sprouted Bengal gram for the initial seven post-operative days, after which the sutures were removed. Analgesia was given with buprenorphine (Bupregesic, Neon Laboratories Ltd., Thane, India) injected subcutaneously in a dose of 0.05mg/kg body weight twice daily, and meloxicam (Melonex, Intas pharmaceuticals Ltd., Ahmedabad, India) injected subcutaneously in a dose of 1mg/kg body weight once daily for five days. Ceftriaxone (Finecef, Abott Healthcare Pvt. Ltd, Thane, India) injection was given at the dose of 15mg/Kg subcutaneously, once daily for five days. Humane endpoints applied to the study were enlisted in detail and all the personnel working with animals were made aware of the objective type of end points. It was decided to euthanize any animals if they lost over 20% of body weight in comparison to the base line body weight. Animals were handled twice daily, and in animals which had lost their normal mobility due to paraplegia, while performing manual evacuation of urine, it was decided to intervene with antibiotics if pus was observed in their urine. The self-grooming of animals was observed and in case of self-mutilation of any body parts, it was decided to euthanize the animals and to exclude the data from those animals in the study. However, the study was concluded eventless with no mortalities or exclusions owing to the application of humane endpoints recorded.

### Basso Beattie Bresnahan (BBB) score

Post-surgical motor function assessment was performed using BBB scoring, as previously described by Basso *et al* [12] on Day 1, 7, 14, 21and 28 post-operatively. Scores were compared between the two types of surgical approaches described above.

### Body weight measurements

Body weights recorded on Day 1, 7, 14, 21 and 28 post-operatively were compared to the baseline body weight, which was recorded immediately prior to anesthesia (Day 0) for each animal.

### RGS score

The animals were habituated by placing them daily in the RGS assessment box for five minutes daily for seven days during the acclimatization period. RGS baseline was obtained on the day of surgery, prior to the initiation of anaesthesia. Two digital video cameras (Sony HDR-CX405, Tokyo, Japan) were placed at the shorter ends of the assessment box–an acrylic box of dimensions 25x12x12 cms (E-Z system corporation, Palmer, PA)–with longitudinal sides completely obscured using opaque black plastic sheets. The box was filled with sterile corncob bedding and the animal was left in the box with top covered with SS 304 grill and was video recorded for 15 minutes. The animals were returned to their home cage immediately after the RGS video recordings. RGS was recorded immediately prior to anesthesia (Day 0) for each animal, which was the baseline RGS and then on Day 1, Day 7, Day 14, Day 21 and Day 28 post-operatively. Five still images were captured at three-minute intervals from each video and was analysed for the four action units as previously described [17]. The first among the four action units of the RGS is orbital tightening, where rats in pain display a narrowing of the orbital area, manifesting as either (partial or complete) eye closure or eye squeezing. The second action unit is nose/cheek flattening, where rats in pain display successively less bulging of the nose and cheek, with eventual absence of the crease between the cheek and whisker pads. Ear changes is the third action unit where the ears of rats in pain tend to fold, curl and angle forwards or outwards, resulting in a pointed shape. The space between the ears may appear wider. Finally, the fourth action unit observed is whisker changes, where the whiskers of in pain move forward (away from the face) from the baseline position, and tend to bunch, giving the appearance of whiskers standing on end [17]. The average RGS score was noted for each animal and the difference in post-operative RGS scores from baseline scores at different time points between the groups were compared.

### Resting time and rearing behaviour

The animals were acclimatized in their observation cage (dimensions 65 X 50 X 20 cm) for five minutes daily for seven days before the procedures started. The resting time of the operated rats was assessed Days 1, 7, 14, 21 and 28 post-operatively, from video recordings obtained after leaving the animals in the observation cage. Resting time as well as the rearing score was assessed from 5 minute video clips, and was compared between groups at Days 1, 7, 14, 21 and 28 post-operatively. Rearing in SCI animals was classed into low rearing and high rearing. Low rearing involved extension of neck and forelimbs to involve in an exploratory rearing posture. High rearing involved complete rearing either with the support of the cage walls or independently on the animal’s hind limbs.

### Euthanasia and histopathology of tissues

The animals were anesthetised using xylazine and ketamine at a dose of 5 mg/kg and 80mg/kg body weight and euthanised using over dosage of 0.5% thiopentone sodium (Thiosol, Neon Laboratories Limited, Mumbai, India) by intraperitoneal injection. Vertebral column with spinal cord at thoracic and lumbar region was dissected out and fixed in 10% neutral buffer formalin for 48 hours. Decalcification was made in 12.5% ethylene diamine tetra acetic acid (EDTA) with frequent checking for decalcification of bone and changing of decalcifying solution when necessary. Grossing was done after which the tissue was washed in water and dehydrated through graded alcohol series. Then, the tissue was cleared in xylene and was embedded in paraffin wax. Sections of 5 μm thickness were cut and stained with haematoxylin and eosin (H&E). Mounted and cover-slipped slides were observed under a trinocular transmitted light microscope (Nikon E600, Nikon, Japan). Microphotographs were captured using a colour camera (Nikon DS-Ri1, Nikon, Japan) attached to the microscope. The histopathology sections were compared and scored for the extent of damage in the spinal cord. Percentage of damage of spinal cord tissue was recorded and compared between groups.

### Statistical analysis

Data are presented as scatter plots with each dot representing one individual animal, the horizontal line representing the mean and the whiskers the standard deviation, and analysed using GraphPad Prism 8.0.2. Each individual animal was treated as an experimental unit. ANOVA was used to analyse normally distributed multiple comparisons followed by Tukey’s multiple post-hoc comparison test. For the data which was not normally distributed, Kruskal-Wallis test was applied to compare more than two groups and Dunn’s multiple comparison test was used to assess the pairwise differences. Since the main focus of this study was to compare differences between the two surgical techniques (CONV and DBA) within the LAM and SCI groups respectively, statistical comparisons between CONV-SCI and DBA-LAM groups are not presented in the figures. Normality of data was tested using Kolmogorov-Smirnov test for all the cases. *P* < 0.05 was considered statistically significant.

## Results

The time taken to complete the procedures did not show any difference between the groups. It was observed that 29.166±7.1 minutes was required to complete the procedures in CONV-LAM group whereas in DBA-LAM group it was 29.83±6.2 minutes. For the CONV-SCI and DBA-SCI group, 26.135±1.35 minutes and 27.3±1.56 minutes was required. The measurements of laminectomy wound showed an overall dimension of 10.63±3.43 mm^2^ for CONV-LAM and 13.22±5.22 mm^2^ for DBA-LAM animals, where as it was 9.302±2.46 mm^2^ and 8.167±2.218 mm^2^ for the CONV-SCI and DBA-SCI groups respectively.There were no statistically significant differences in wound dimension between the groups. The term ‘wound’ refers specifically to the laminectomised area, and not to the general wounding of tissue due to the surgical incision and similar. Histopathology data revealed that the percentage of damage caused due to laminectomy did not vary between techniques in any of the groups.

### BBB score

**Fig 2:**
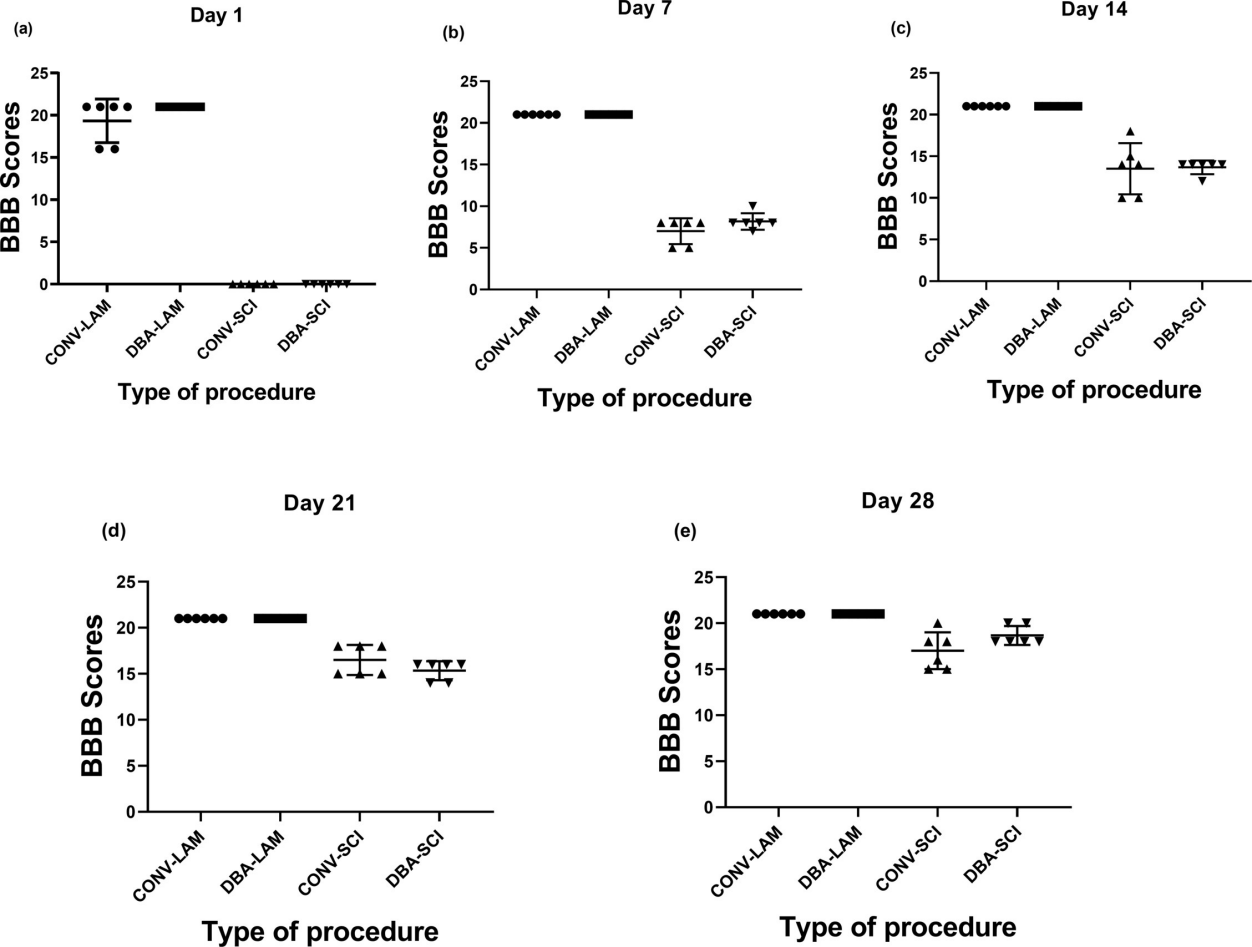
Post-operative BBB score comparison. BBB scores of rats (n = 6 per group) subjected to laminectomy using conventional (CONV-LAM) or motorized dental burr assisted (DBA-LAM) technique at, or subjected to spinal cord injury induced using conventional (CONV-SCI) or dental burr assisted (DBA-SCI) technique during the study period. Scores differed between groups on Day 1 (a), Day 7 (b), Day 14 (c), (Day 21 (d), and Day 28 (e) postoperatively as determined by Kruskal-Wallis test with Dunn’s multiple comparisons. Data are presented as scatter plot with each dot representing one individual animal, the horizontal line the mean and the whiskers the standard deviation.

The motor coordination and function of animals that underwent laminectomy through either of the two techniques were normal and didn’t show any reduction in BBB score. Following the induction of SCI by a severe contusion injury at T10-T11 level, SCI animals in both the groups had an initial BBB score of 0 on Day 1. There were significant differences in the BBB scores between LAM and SCI groups on Day 1 (, Kruskal-Wallis statistic = 21.81, *P*<0.0001) (Fig 2A), Day 7 (Kruskal-Wallis statistic = 20.89, *P*<0.0001) (Fig 2B), Day 14 (Kruskal-Wallis statistic = 20.32, *P*<0.0001) (Fig 2C), Day 21 (Kruskal-Wallis statistic = 20.2, *P* = 0.0002) (Fig 2D) and Day 28 (Kruskal-Wallis statistic = 20.76, *P* = 0.0001) (Fig 2E) postoperatively. Pairwise comparisons are presented in detail in Fig 1A–1E. Apart from the differences found between LAM and SCI groups, there were no differences between the CONV and DBS techniques neither in LAM nor in SCI groups. A progressive improvement of hind limb motor functional ability and BBB scores up to 17±1.91 in CONV-SCI and 18.67±0.99 in DBA-SCI was noticed at Day 28 postoperatively (Fig 2E).

### Body weights

**Fig 3:**
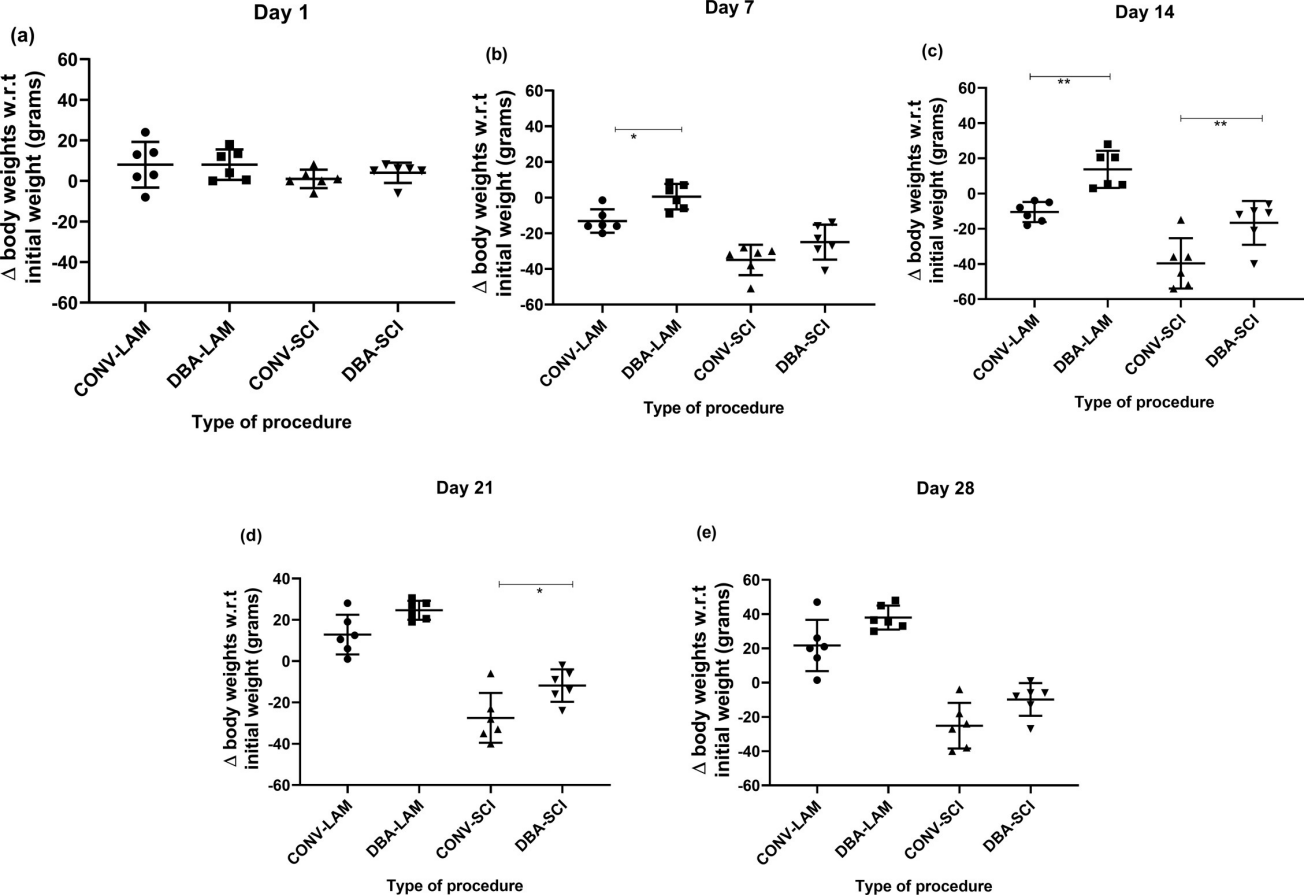
Post-operative body weights comparison. Postoperative weight fluctuation (compared to the initial body weights) in rats (n = 6 per group) subjected to laminectomy using conventional (CONV-LAM) or motorized dental burr assisted (DBA-LAM) technique at, or subjected to spinal cord injury induced using conventional (CONV-SCI) or dental burr assisted (DBA-SCI) technique during the study period on Day 1 (a), Day 7 (b), Day 14 (c), Day 21 (d) and Day 28 (e) post-operatively. Differences were analysed by ANOVA with Tukey’s multiple comparisons. Data are presented as scatter plot with each dot representing one individual animal, the horizontal line the mean and the whiskers the standard deviation. * = P<0.05, ** = P<0.01.

Body weights did not fluctuate from the baseline value on day 1 (Fig 3A), whereas it differed significantly between groups on post-operative days 7 (F(3,20) = 21.39, *P* = <0.0001) (Fig 3B), 14 (F(3,20) = 23, *P* = <0.0001) (Fig 3C), 21 (F(3,20) = 41.63, *P* = <0.0001) (Fig 3D) and 28 (F(3,20) = 36.75, *P* = <0.0001) (Fig 3E), as determined by ANOVA with Dunn’s multiple comparisons. DBA-LAM showed significant weight gain on days 7 (*P* = 0.039) and 14 (*P* = 0.0064) in comparison with the CONV-LAM group. On days 14 (*P* = 0.0099) and 21 (*P* = 0.0306) (Fig 2D), CONV-SCI group showed significant weight loss in comparison to DBA-SCI group.

### RGS score

**Fig 4:**
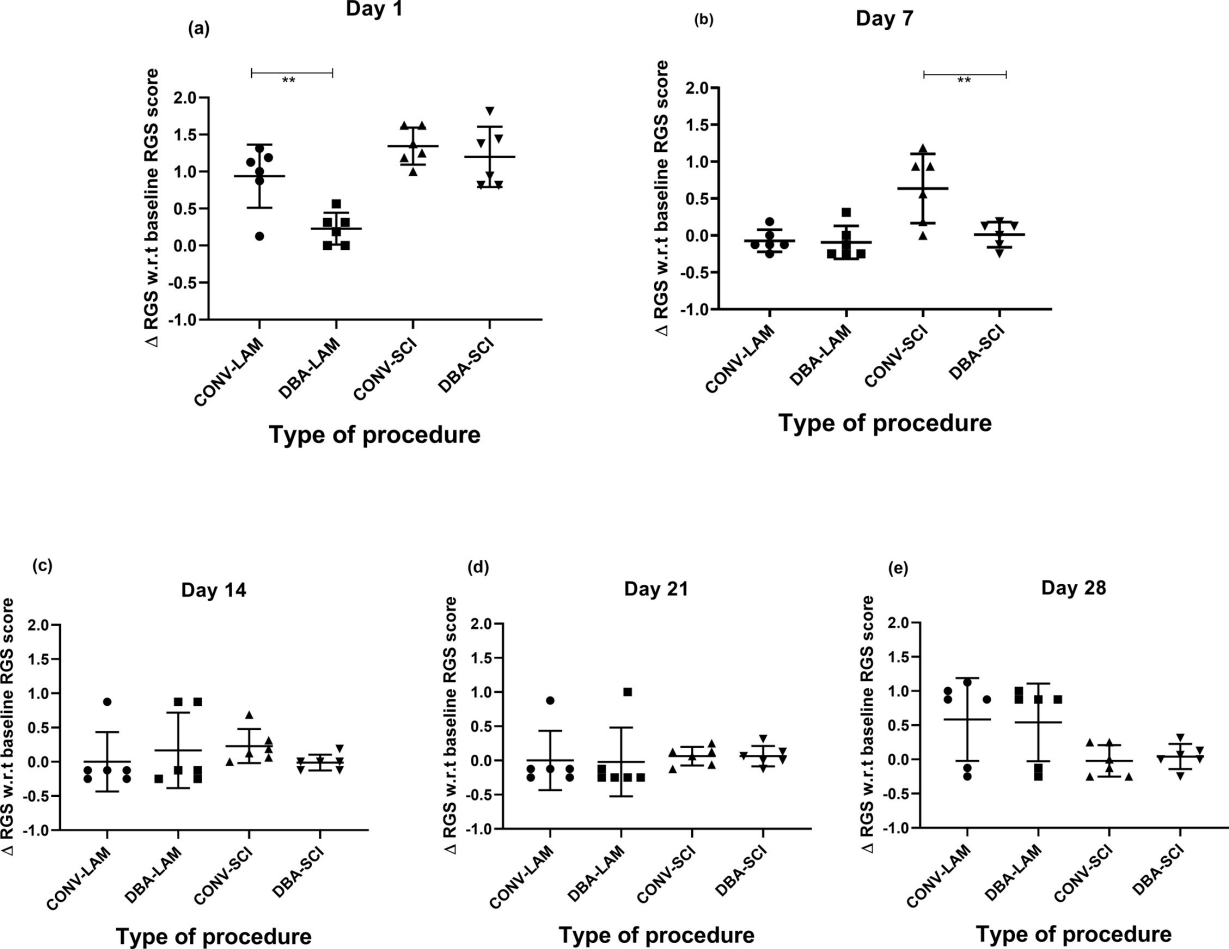
Post-operative RGS scores comparison. Change of RGS scores from baseline values of rats (n = 6 per group) subjected to laminectomy using conventional (CONV-LAM) or motorized dental burr assisted (DBA-LAM) technique at, or subjected to spinal cord injury induced using conventional (CONV-SCI) or dental burr assisted (DBA-SCI) technique during the study period. RGS scores differed significantly on Day 1 (a) and Day 7 (b) post-operatively between groups, as determined by Kruskal-Wallis test with Dunn’s multiple comparisons. On Day 14 (c) postoperatively, ANOVA with Tukey’s multiple comparisons revealed a significant difference in RGS score in CONV-SCI group compared to the CONV-LAM group. No differences between groups were observed Day 21 (d) or Day 28 (e). Data are presented as scatter plot with each dot representing one individual animal, the horizontal line the mean and the whiskers the standard deviation. ** = P<0.01.

The baseline RGS values of CONV-LAM (0.1875±0.06), DBA-LAM (0.1786±0.06), CONV-SCI (0.1±0.12) and DBA-SCI (0.1±0.12) did not differ between groups. The groups differed in RGS scores significantly on Day 1 (F(3,20) = 12.88, *P* = <0.0001) (Fig 4A) and 7 (F(3,20) = 9.005, *P* = 0.0006) (Fig 4B) postoperatively. Pairwise comparisons are presented in detail in Fig 4A–4E. The RGS score was significantly lower in DBA-LAM in comparison with CONV-LAM on Day 1 (*P*< 0.001) (Fig 4A) post-operatively. Similarly, the RGS score was significantly lower in DBA-SCI in comparison with CONV- SCI on Day 7 (*P*< = 0.0054) (Fig 4B) post-operatively. RGS scores did not differ between groups on Days 14, 21 and 28 (Fig 4C–4E) post-operatively.

### Resting time and rearing behaviour

**Fig 5:**
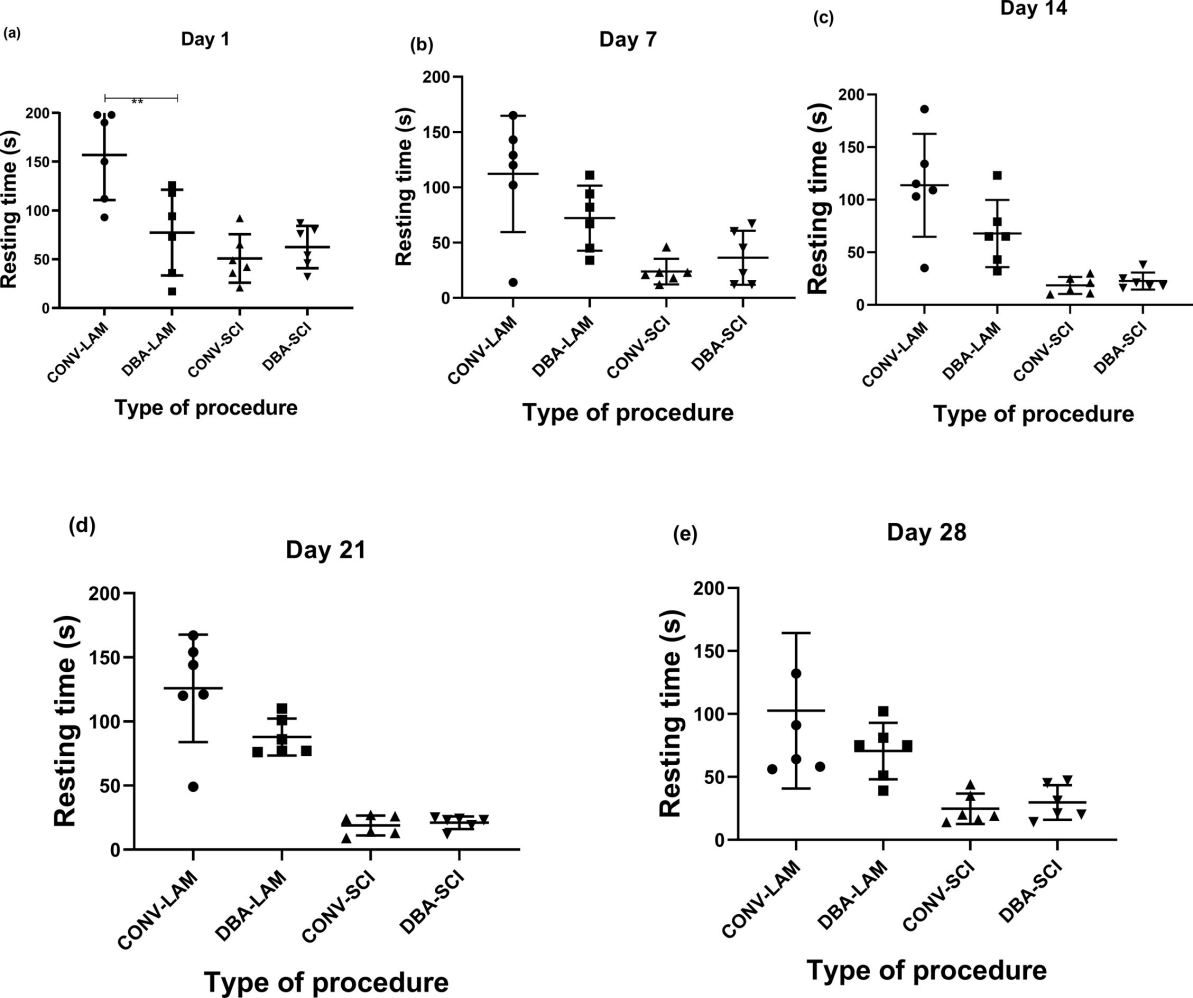
Post-operative resting time comparison. Resting time of rats (n = 6 per group) subjected to laminectomy using conventional (CONV-LAM) or motorized dental burr assisted (DBA-LAM) technique at, or subjected to spinal cord injury induced using conventional (CONV-SCI) or dental burr assisted (DBA-SCI) technique during the study period. Resting time differed significantly between groups on Day 1 (a), Day 14 (c) and Day 28 (e), as determined by ANOVA with Tukey’s multiple comparisons, and on Day 7 (b) and Day 21 (d) postoperatively, as determined by Kruskal-Wallis test with Dunn’s multiple comparisons. Data are presented as scatter plot with each dot representing one individual animal, the horizontal line the mean and the whiskers the standard deviation. ** = P<0.01.

The most prominent finding from the analysis of resting time was that the LAM groups showed more resting time in comparison with the SCI groups. On Day 1, groups differed significantly in resting time (F(3,20) = 10.69, *P* = 0.0002) and the CONV-LAM showed significantly more resting time than the DBA-LAM group (*P*<0.001) (Fig 5A). Further, the groups differed at Day 7 (Kruskal-Wallis statistic = 10.99, *P* = 0.0118) (Fig 5B), Day 14 (F(3,20) = 13.51, *P*<0.0001) (Fig 5C), Day 21 (Kruskal-Wallis Statistic = 18.27, *P*<0.0004,) (Fig 5D), and Day 28 (F(3,20) = 6.959, *P* = 0.0022) (Fig 5E) postoperatively, but there were no differences between SCI groups nor between LAM groups other than the one mentioned on Day 1.

**Fig 6:**
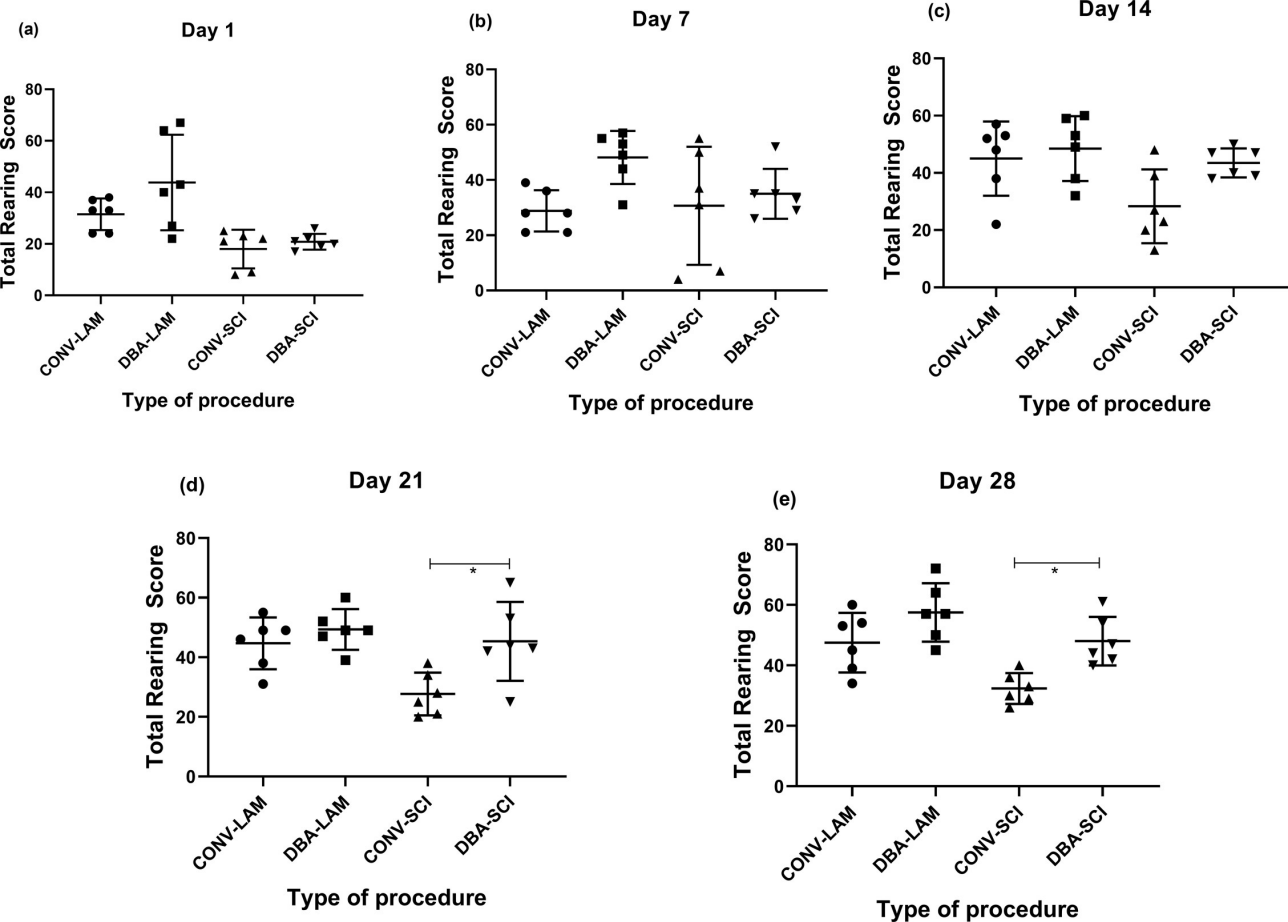
Post-operative rearing score comparison. Rearing scores of rats (n = 6 per group) subjected to laminectomy using conventional (CONV-LAM) or motorized dental burr assisted (DBA-LAM) technique at, or subjected to spinal cord injury induced using conventional (CONV-SCI) or dental burr assisted (DBA-SCI) technique during the study period. Rearing scores differed significantly between groups on Day 1 (a), 14 (c), 21(d) and 28 (e) postoperatively. Differences were analysed by ANOVA with Tukey’s multiple comparisons. No differences between groups were observed Day 14 (b). Data are presented as scatter plot with each dot representing one individual animal, the horizontal line the mean and the whiskers the standard deviation. * = P<0.05.

The groups differed in total rearing scores on Day 1(F (3,20) = 7.393, *P* = 0.0016) where the DBA-LAM group showed more frequent rearing in comparison with the CONV-SCI (*P*<0.01) and DBA-SCI (*P*<0.05) (Fig 6A). The groups did not differ in rearing scores on Day 7 (Fig 6B). It was noted that the groups differed at Day 14 (F(3,20) = 3.887, *P* = 0.0244) (Fig 6C), Day 21 (F(3,20) = 6.353, *P* = 0.0033) (Fig 6D) and Day 28 (F(3,20) = 9.239, *P* = 0.0005) (Fig 6E) and importantly, DBA-SCI exhibited better rearing scores in comparison to CONV-SCI on Day 21 (*P*<0.05) and Day 28 (*P*<0.05).

**Fig 7:**
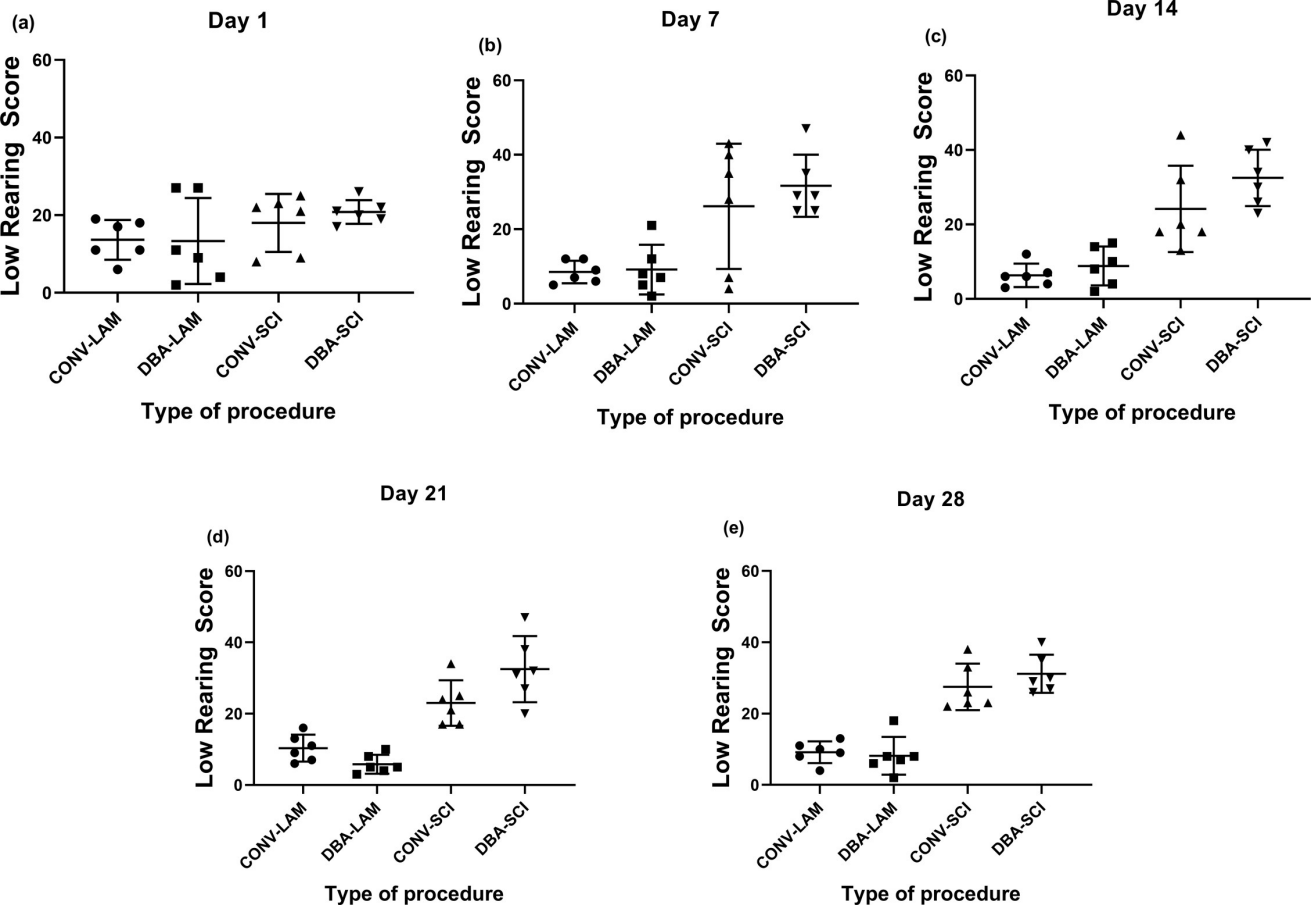
Post-operative low-rearing scores comparison. Low rearing scores of rats (n = 6 per group) subjected to laminectomy using conventional (CONV-LAM) or motorized dental burr assisted (DBA-LAM) technique at, or subjected to spinal cord injury induced using conventional (CONV-SCI) or dental burr assisted (DBA-SCI) technique during the study period. Low rearing scores differed significantly between groups on Day 7 (b), 14 (c), 21(d), as determined by ANOVA with Tukey’s multiple comparisons, and on Day 28 (e) postoperatively, as determined by Kruskal-Wallis test with Dunn’s multiple comparisons. Data are presented as scatter plot with each dot representing one individual animal, the horizontal line the mean and the whiskers the standard deviation.

The groups did not differ in low rearing scores on Day 1 (Fig 7A). The groups differed significantly in low rearing on Day 7 (F(3,20) = 8.240, *P* = 0.0009) (Fig 7B), Day 14 F(3,20) = 16.34, *P*<0.0001) (Fig 7C), Day 21 F(3,20) = 23.97, *P*<0.0001) (Fig 7D) and Day 28 (Kruskal-Wallis statistic = 18.14, *P* = 0.0004) (Fig 7E) postoperatively. Low rearing was significantly more frequent in the SCI groups, than in the LAM groups.

**Fig 8:**
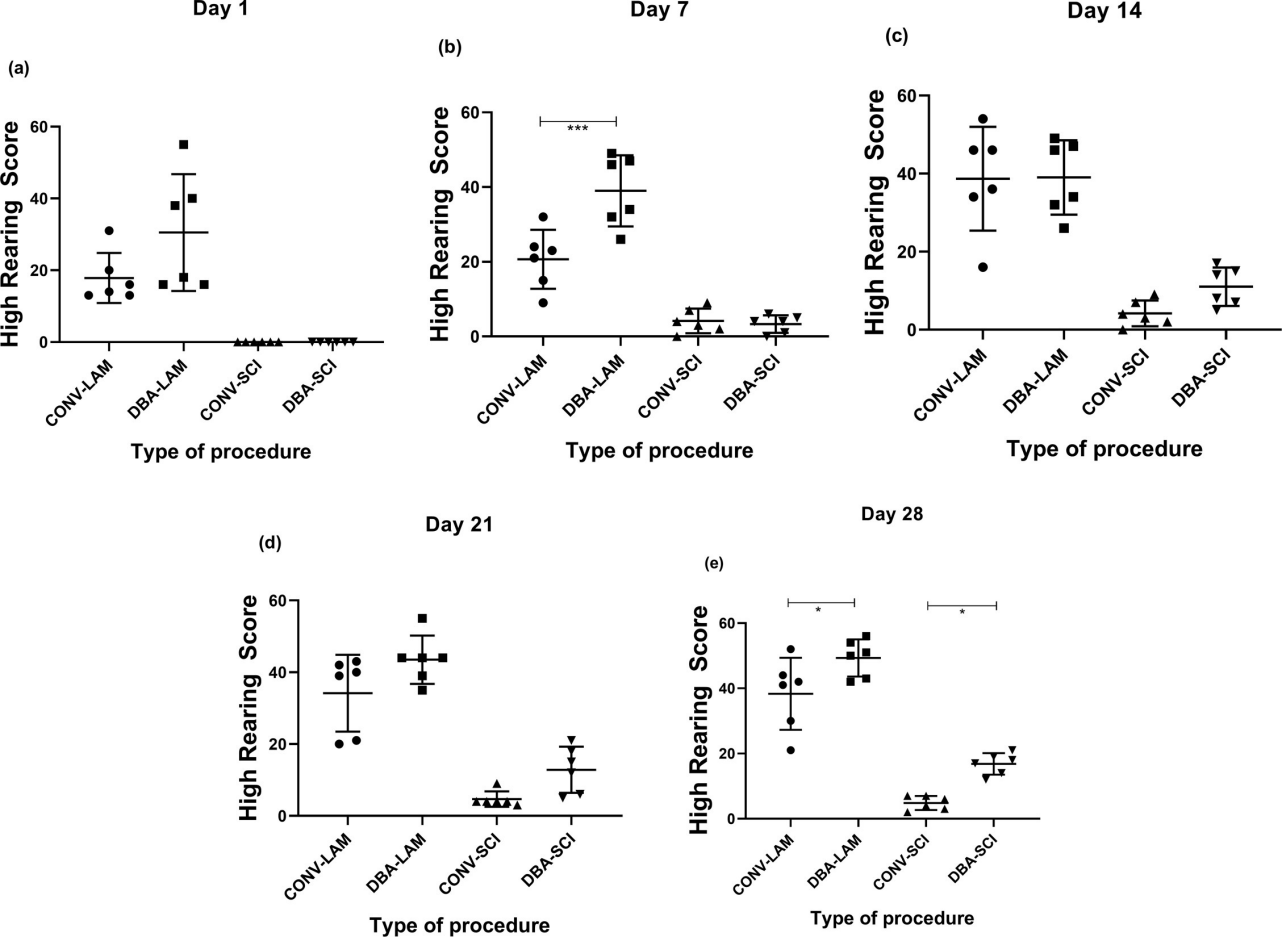
Post-operative high scores comparison. High rearing scores of rats (n = 6 per group) subjected to laminectomy using conventional (CONV-LAM) or motorized dental burr assisted (DBA-LAM) technique at, or subjected to spinal cord injury induced using conventional (CONV-SCI) or dental burr assisted (DBA-SCI) technique during the study period. High rearing scores differed significantly between groups on Day 1 (a) and 21 (d) post-operatively between groups, as determined by Kruskal-Wallis test with Dunn’s multiple comparisons. On Day 7 (b), 14 (c) and 28 (e) postoperatively, ANOVA with Tukey’s multiple comparisons revealed a significant difference between groups. Data are presented as scatter plot with each dot representing one individual animal, the horizontal line the mean and the whiskers the standard deviation. * = P<0.05, *** = P<0.001.

The groups differed significantly in high rearing on Day 1 (Kruskal-Wallis statistic = 20.71, *P* = 0.0001) (Fig 8A), Day 7 (F(3,20) = 39.87, *P*<0.0001) (Fig 8B), Day 14 (F(3,20) = 26.35, *P*<0.0001) (Fig 8C), Day 21 (Kruskal-Wallis statistic = 19.42, *P* = 0.0002) (Fig 8D) and Day 28 (F(3,20) = 57.46, *P*<0.0001) (Fig 8E) postoperatively. High rearing was more frequent in the LAM groups than in the SCI groups. It is important to note that on Day 7 (P<0.001) and Day 28 (P<0.05), DBA-LAM group exhibited more high rearing score in comparison with the CONV-LAM group and on Day 28 (P<0.05), DBA-SCI exhibited more high rearing score than CONV-SCI.

## Discussion

Behavioural tests employed in the assessment of SCI in rat models mainly focus on motor abilities, which includes grid walking and body balancing tests [13,19], gait parameters [20], leg movements [21], bipedal walking [21,22], swimming and wading [23,24]. The BBB scoring test has been used in more than thousand publications till date [5]. In addition, motor tests like inclined plane, limb hanging test, limb grip strength test, forelimb asymmetry test, rearing test and food pellet reaching test are being employed to assess the recovery and functional status of the SCI rat model [13]. Several sensory and sensory-motor tests, reflex response-based tests autonomic tests like urinary bladder function, erection tests, telemetric monitoring and autonomic dysreflexia testing [13], as well as tests to observe increasing motor activity of animals housed in enriched environment [25], tread-mill running wheel training, swimming and robot-assisted hind limb extension are employed to assess functional recovery in SCI models.

Combined behaviour score [26] employs a battery of tests including toe spread reflex, pacing reflex, withdrawal in response to stimulus, righting and hot plate tests, coordination between hindlimb and forelimb, weight support during walking, swimming and standing on an inclined plane to make a combined score to assess the SCI rats. The combined behavioural scoring system has–similar to the BBB scoring–the disadvantage of being non-linear [13], and so, the animals with identical final score may represent two different levels of recovery [13]. Further, by employing only motor tests to compare between groups the welfare levels, adequacy of analgesia as well as its effect on the outcome of the animal model is being overlooked.

As most studies applying the SCI contusion model have focussed on the above mentioned parameters, there is a lack of studies investigating welfare aspects related to the model. This, in turn, has made development of novel techniques for induction and validation of the model more difficult. Therefore, the present study was designed to investigate the effect of a novel surgical technique compared to the conventional method on various parameters that could be relevant to the welfare state of the animal, and to investigate whether this novel technique would impair the quality of the model by changes in the BBB scores induced by the SCI. The time taken for completion as well as the wound dimensions did not show any differences between groups indicating no additional time requirement with comparable wound size for operation. BBB scores did not differ between techniques. This means that the novel surgical technique could be implemented among scientists without changing the scientific outcome, while simultaneously minimizing potential suffering in the animals. Body weight comparisons during post procedural recovery have previously been shown to be an effective tool in assessment of welfare and different effects of techniques in rodents [27]. In the current study, significant improvement in the postoperative body weight gain in the DBA-LAM group was observed, as well as a marked reduction in postoperative weight loss in the DBA-SCI group in comparison to their conventional counterparts.

The histopathology analysis did not demonstrate any differences between the groups. It cannot be determined whether this is due to a true non-existing difference, or due to that the specific staining method applied (H&E) was insufficient for detecting any differences. Therefore we are planning to incorporate other staining methods such as Nissle’s or Eriochrome CR, to further elucidate potential histological changes between the techniques.

RGS is a tool widely used to assess levels of post-procedural pain in rats on various occasions [28,29,30]. The RGS scoring applied in the present study demonstrates that pain can be present in the animals with SCI induced by conventional laminectomy until Day 7 post-operatively. This points towards the importance of considering an effective post-operative analgesic regimen of adequate duration when inducing SCI using conventional surgical technique. The significant reduction in the RGS scores on Day 1 in DBA-LAM and on Day 7 in the DBA-SCI group with respect to their respective counterparts suggests the procedural advantage of motorising the laminectomy procedure when surgically inducing SCI in rats. It is noteworthy that there is apparently more variability in RGS scores in the CONV-SCI group in comparison to the DBA-SCI group on Day 7. This could be due to that the DBA technique causes more standardised and uniform delivery of shearing forces, which in turn could cause less severe and more uniform trauma in this group, leading to less variation in the response. Meanwhile, the high variation could be problematic, since it could create a lower statistical power to the experiment, and thus leading to increased occurrence of type II errors, masking actual differences between groups. This underlines the importance of not relying on one single parameter when evaluating the levels of pain and welfare, but including a battery of tests as applied in the present study.

Many studies using the rat SCI model appear to overlook the use of postoperative analgesia, considering the lack of reporting of the use of analgesic regimens in many publications describing this model [31,32,33,34,35]. Where analgesic regimen is reported, variable dosages and durations of administration of analgesics are described, such as acetaminophen 65 mg/kg orally, only if the rat shows the sign of self-mutilation [36]; buprenorphine 0.3mg/kg once daily s/c for two postoperative days [37]; 1.5 ml (0.006 mg/ml) buprenorphine twice daily subcutaneously for first 24–48 hours [38]; and buprenorphine 0.01mg/kg b.i.d. for three post-operative days [39]. It is unknown whether these analgesic regimens actually provide sufficient analgesia, due to the fact that most of these studies are focused on therapeutic development, and it is unclear to what extent the investigators have incorporated techniques to assess the status of pain as a component of their therapeutic efficacy. Our study confirms that RGS scoring could be of great use in a postoperative battery of tests, to determine the analgesic efficacy and to decide upon relevant dose and duration of post-operative analgesia.

The resting time was longest in the CONV-LAM animals, followed by DBA-LAM animals. The DBA-SCI and CONV-SCI groups showed more activity in comparison to the LAM groups, in particular to the CONV-LAM group. The increased activity in the SCI-animals during the day time may be due to behavioural and functional distress. However, there are also differences between the CONV-LAM and the DBA-LAM groups, for which we have no explanation, why more studies are needed to elucidate this. It is worth pointing out that the activities were measured during the light period of the light:dark cycle, which is the time where the animals are most inactive.

The home cage activity during the dark phase should also be studied in order to generate a better understanding on the impact of these techniques on the welfare of the animals. It has been reported already that rearing can be used to assess spontaneous hind limb activity in SCI rats [40]. It is worthwhile to note that the study demonstrated considerably more low-rearing behaviour in SCI animals whereas LAM animals performed more high-rearing. Total rearing score on Day 21 and 28 and high rearing score on Day 28 post-operatively in DBA-SCI was higher in comparison to the CONV-SCI which indicates the utility of studying the rearing scores to evaluate the functional recovery of the model.

The impact of the surgical methodology for inducing SCI by laminectomy has, to our knowledge, been sparsely investigated. From our work, it could be summarised that inducing SCI using a motorised dental burr for facilitating the laminectomy could bring refinement to the animal model. Motorising the laminectomy procedure is likely to have lowered the jerky movements to the tissue *ad nexa* and thereby decreased the nociceptive input from the tissue and result in an overall improvement of the model. Even though assisting laminectomy procedure with a dental burr could be recommended, it must be strictly underlined that adequate pre-training and experience is a necessity to bring out best results. Motorised surgical approach could damage the soft tissues if the bone is cut through, without adequate control of the procedure. In recent years there has been a growing body of clinical evidence that has demonstrated neuroprotective effects of estrogen and progesterone, and clinical reports are supported by experimental preclinical evidence of the neuroprotective effects of estrogen in animal models [41]. Thus, the outcomes of behavioural, welfare and functional parameters may differ if using male animals instead.

In conclusion, inducing a SCI rat model adopting the motorized dental burr method described in this study is a better option with less impaired wellbeing in the animals, compared to the conventional method. Supplementing the BBB score with a battery of tests for welfare assessment, like postoperative body weight changes, RGS, and percentage of activity and rest, could assist investigators to employ adequate post-operative analgesia throughout the painful phase of the SCI, without compromising the model outcome.
